# Primary central nervous system lymphoma and tumefactive demyelinating lesions in multiple sclerosis: a retrospective observational cohort study

**DOI:** 10.3389/fonc.2026.1763680

**Published:** 2026-05-29

**Authors:** Yushan Chen, Lvying Shen, Shaoqiang Chen, Shunyong Zheng, Jianmin Xie, Shuishun Zheng

**Affiliations:** 1 Radiology Department, Zhangzhou Affiliated Hospital of Fujian Medical University, Zhangzhou, Fujian, China; 2 Hematology Department, Zhangzhou Affiliated Hospital of Fujian Medical University, Zhangzhou, Fujian, China; 3 Department of Human Anatomy, Fujian Medical University, Fuzhou, China; 4 Department of Imaging, Zhangzhou Affiliated Hospital of Fujian Medical University, Zhangzhou, China; 5 Neurosurgery Department, Fujian Zhangzhou Traditional Chinese Medicine Hospital, Zhangzhou, Fujian, China; 6 Neurosurgery Department, Zhangzhou Affiliated Hospital of Fujian Medical University, Zhangzhou, Fujian, China

**Keywords:** diagnosis, neuroimaging, pathology, primary central nervous system lymphoma, tumefactive demyelinating lesions

## Abstract

**Background:**

Differentiating primary central nervous system lymphoma (PCNSL) from tumefactive demyelinating lesions (TDLs) remains a major diagnostic challenge.

**Objective:**

Our study aimed to compare the differences in clinical observations, neuroimaging data, and pathological features between the PCNSL and TDLs, as well as their potential biomarkers.

**Method:**

This was a retrospective observational cohort study of 183 patients (97 PCNSL and 86 TDLs). Baseline clinical data were collected. Neuroimaging features were determined by MRI. Pathological characteristics were evaluated by using hematoxylin-eosin (HE) staining, and immunochemical staining of CD3 and CD20. Cerebrospinal fluid (CSF) and serum biomarkers were detected by using the commercial protein assay kit and the enzyme-linked immunosorbent assay.

**Results:**

Patients with PCNSL had an older onset age, with the mean age being 59 ± 10 years in the PCNSL group and 36 ± 8 years in the TDL group (p < 0.001). Upon MRI scans, brain lesions in PCNSL showed U-shaped hyperintense, while lesions in TDLs showed slightly diffuse hyperintense on diffusion-weighted imaging (DWI) sequence. PCNSL lesions showed obvious hyperintense and perifocal edema, while TDL lesions showed isointense with partial perifocal edema on fluid attenuated inversion recovery (FLAIR) sequences. CD20 was strongly positive, while CD3 was negative in PCNSL tissues, which was different from a lower CD20 and stronger CD3 levels in TDLs. IL-6 and IL-10 levels was higher in the CSF of PCNSL than TDLs.

**Conclusion:**

Careful and comprehensive evaluation of clinical, neuroimaging, and pathological features, and CSF IL-6 and IL-10 levels facilitates the differential diagnosis betweenPCNSL and TDLs.

## 1 Introduction

Primary central nervous system lymphoma (PCNSL) is a rare non-Hodgkin lymphoma with high malignancy, accounting for 3% of primary brain tumors (1). PCNSL does not affect lymph nodes outside the central nervous system (2), and it has a poorer prognosis than peripheral lymphoma (3). The clinical features of PCNSL are mostly headache, weakness, and other symptoms of intracranial hypertension, which are similar to those of other CNS tumors (4). Thus, the diagnostic accuracy of PCNSL is low because of the lack of specific clinical and neuroimaging features (4). Some PCNSL cases could take the form of “sentinel demyelination,” characterized by histologically confirmed demyelinating inflammatory lesions, which are the features of other nonneoplastic diseases, such as multiple sclerosis (MS) or acute disseminated encephalomyelitis (ADEM) (5).

The demyelinating inflammatory lesions in the central nervous system are also called demyelinating pseudotumor (DPT) or tumefactive demyelinating lesion (TDLs), exhibiting space-occupying lesions on neuroimaging, which are similar to those of brain tumors (6). The clinical and pathological aspects of sentinel demyelination of PCNSL and TDL are similar, with contrast-enhancing white matter lesions and relapsing and remitting symptoms. They also show signs that improve with steroid therapy, leading to diagnostic confusion. Although biopsy of the suspected lesion is the gold standard for diagnosis (7), biopsy of the sentinel lesions in the early phase of PCNSL is still of high misdiagnosis rate, especially in the setting of prior corticosteroid administration (8, 9). The treatment of PCNSL, mainly surgery, is very different from that of TDL, which can benefit from the corticosteroid therapy and require no surgery. Patients with TDLs may undergo the unnecessary surgical resection or radiotherapy due to misdiagnosing as PCNSL, while some patients with PCNSL may fail to receive appropriate treatment in a timely manner for misdiagnosing as TDLs, resulting in worse prognosis (10, 11). Thus, it’s of vital importance to investigate the potential differences between PCNSL and TDLs.

In the present study, we collected data from patients with PCNSL and TDLs and reported the differences between PCNSL and TDLs in clinical features, neuroimaging and pathological characteristics, and biomarkers.

## 2 Methods

### 2.1 Patients

A total of 97 patients with PCNSL and 86 patients with TDL, treated in the Zhangzhou Affiliated Hospital of Fujian medical University from 2012 to 2022, were included in the present study. The research was approved by the ethics committee of Zhangzhou Affiliated Hospital of Fujian medical University. All the patients confirmed and signed the written informed consent.

The inclusion criteria were as follows (1): patient age≥18 (2). PCNSL confirmed by pathology (3). TDL diagnosed by pathology or the corresponding criteria: demyelinating lesions measuring ≥20 mm in diameter, or lesions of 5–20 mm with mass effect that mimic tumor-like space-occupying lesions and exhibit characteristic radiological features (12–14) (4); available cerebral MRI before diagnosis, or the imaging data were relatively complete (including the availability of head CT, MRI scans, and DWI sequences) (5). Clinical data were adequately documented and complete. The exclusion criteria were as follows (1): Presence of HIV or EBV positivity, or comorbidities with other known immunodeficiency diseases (2). Inability to tolerate lumbar puncture examination (3). Prior history of other lymphoma or malignant tumors.

### 2.2 Neuroimaging

Magnetic resonance imaging (MRI) diagnosis: The patient was diagnosed with a 3.0 superconducting MR machine (General Electric, Milwaukee, WI, USA). The parameters are set as previously described (15) with minor modification. For T1-weight imaging (T1WI), the repetition time (TR) was set to 2,415 ms, echo time (TE) to 13.9 ms, and 6-mm slice thickness with a 2-mm interslice gap. For T2-weighted imagining (T2WI), the TR was set to 4,660 ms, TE to 110 ms, and 6-mm slice thickness with a 2-mm interslice gap. FLAIR imaging was used the parameter as follows: TR 9,000 ms; TE 120 ms; TI 2,200 ms, matrix 256 × 192, field of view (FOV) 240 mm; 6-mm slice thickness with a 2-mm interslice gap. The parameters of diffusion-weighted imaging (DWI) were obtained in the axial plane using echo-planar sequence with the following parameters: TR 7,000ms; TE 83.7 ms, 6-mm slice thickness with a 2-mm interslice gap. Contrast T1WI was used the parameter as follows: TR, 400–600 ms; TE, 6–10 ms; matrix, 256 × 224; field of view (FOV), 240 mm; 6-mm slice thickness with a 2-mm interslice gap. Gadolinium diethylenetriamine pentaacetic acid (Gd-DTPA 0.1 mmol/kg) was used as the contrast material, and the injection rate was 4 ml/s.

### 2.3 Histopathological study

The tumors removed from surgery or biopsy were collected in formalin. The tissue was embedded in paraffin. For hematoxylin-eosin (HE) staining, the prepared tissue slices were dewaxed, hydrated, and stained in hematoxylin solution for 5 min. After differentiated by 1% hydrochloric alcohol for 15s, the slices were washed with water, following with staining by eosin solution for 1 min. Finally, the slices were dehydrated, transparentized, sealed by neutral gum, and observed under an optical microscope (Olympus, Tokyo, Japan). For immunohistochemical (IHC) analysis, the paraffin-embedded sections were dewaxed in xylene and rehydrated in alcohol. Endogenous peroxidase was blocked by 3% H_2_O_2_, and antigen was retrieved through microwave heating. After blocking nonspecific antigen by using 5% BSA at 37 °C for 1 h, the sections were incubated with a specific primary antibody against CD3 (1:1000; Abcam, USA) or CD20 (1:1000; Abcam, USA) at 4 °C overnight. Then the goat anti-rabbit secondary antibody conjugated to horseradish peroxidase (1:5000, Bioworld, USA) were incubated at 37 °C for 1 h, the sections were stained with diaminobenzidine and counterstained with hematoxylin. Representative images were taken using an optical microscope (Olympus, Tokyo, Japan). IHC staining was evaluated based on cytoplasmic or membranous brownish-yellow to brown coloration, which was defined as positive. The proportion of positive cells was scored as follows (16): <10% = score 0 (“Negative”); 11–25% = score 1 (“Low”); 26–50% = score 2 (“Intermediate”); >50% = score 3 (“High”).

### 2.4 Cerebrospinal fluid and serum analyses

Blood of the patients were drawn from elbow venous at a volume, following with centrifugation (3,000 r/min, 10 min) to obtain the serum. CSF was collected at through the lumbar puncture. After centrifuging at 500r/min for 10min, the supernatant was placed in the frozen tube and stored in -80°C refrigerator. The protein level in the CSF was determined by using the protein assay kit (Beyotime, Shanghai, PR China). IL-6 and IL-10 were detected by using the enzyme-linked immunosorbent assay (ELISA) with the commercialized ELISA kit (Lianke Biotechnology, Shanghai, China). Briefly, 100 μl of serum sample was added to a 96-well plate coated with human target protein. Then 100 μl of HRP-conjugated mixed solution was added to each well, and the plate was incubated for 0.5 h at 37 °C. After several washes, the color reaction was developed with the substrate solution and blocked with stop solution. The optical densities were measured at 450 nm. All the experiments were operated strictly in accordance with the manufacturer’s instructions.

### 2.5 Statistical analyses

All statistical analyses were performed using GraphPad (version 9.3.1) and SPSS (version 28.0). The normality of continuous variables was assessed using the Kolmogorov–Smirnov test. Continuous variables are presented as mean ± standard deviation (SD) and were compared between groups using the independent-samples Student’s t-test. Categorical variables are expressed as counts and percentages and were analyzed using the Pearson chi-square test or Fisher’s exact test, as appropriate. To account for multiple comparisons, the Bonferroni correction method was applied, and adjusted p values are reported where applicable. Receiver operating characteristic (ROC) curves were generated to assess the diagnostic efficacy of the relevant indicators for distinguishing PCNSL from TDL. The area under the ROC curve (AUC) with 95% confidence intervals (95% CIs) was calculated, and optimal cut-off values were determined using the Youden’s index, with corresponding sensitivity and specificity reported. A two-sided p value < 0.05 was considered statistically significant.

## 3 Results

### 3.1 Clinical features of PCNSL and TDLs

The clinical features of PCNSL and TDLs are shown in **Table 1**. The onset age of PCNSL patients (59 males and 38 females) was 59.28 ± 10.23 years, with an insidious or slow onset. The initial symptoms of PCNSL included headache in 26 cases (26.8%), weakness in 17 cases (17.5%), numbness in 10 cases (10.3%), reduced vision in 8 cases (8.3%), and limb movement disorder in 8 cases (8.3%). The onset age of TDLs patients (42 males and 44 females) was 36.28 ± 8.11 years, with an acute onset. The initial symptoms of TDLs included headache in 28 cases (32.6%), weakness in 13 cases (15.1%), numbness in 12 cases (14.0%), reduced vision in 7 cases (8.1%), and limb movement disorder in 5 cases (5.8%). Five PCNSL patients underwent more than two biopsies to find tumor cells, among which 3 cases had received glucocorticoid treatment before biopsy. The latter may cause histopathological changes and misdiagnosis. Two TDLs patients were initially diagnosed as “PCNSL” and received gamma knife therapy and glucocorticoid treatment, and their symptoms were relieved. However, the pathological diagnosis was demyelinating disease with radiation encephalopathy because the disease worsened to dementia or loss of functional independence years after gamma knife therapy and glucocorticoid treatment. The onset age of PCNSL patients was older than that of TDLs patients (p<0.01). There was no difference in gender and initial symptoms between PCNSL and TDLs, indicating that these features are not sufficient as a basis for the diagnosis of PCNSL or TDLs.

**Table 1:** The clinical features of PCNSL and TDLs.

Parameter	PCNSL	TDLs	p
Number of subjects	97	86	
Age(years)	59.28 ± 10.23	36.28 ± 8.11	p<0.01
Gender			p>0.05
Male	59	42	
Female	38	44	
Initial symptoms			p>0.05
Headache	26 (26.8%)	28 (32.6%)	
Cognitive dysfunction	17 (17.5%)	10 (11.6%)	
Numbness	10 (10.3%)	12 (14.0%)	
Reduced vision	8 (8.3%)	10 (11.6%)	
Limb movement disorder	8 (8.3%)	5 (5.8%)	

### 3.2 Neuroimaging of PCNSL and TDL

**Figure 1:**
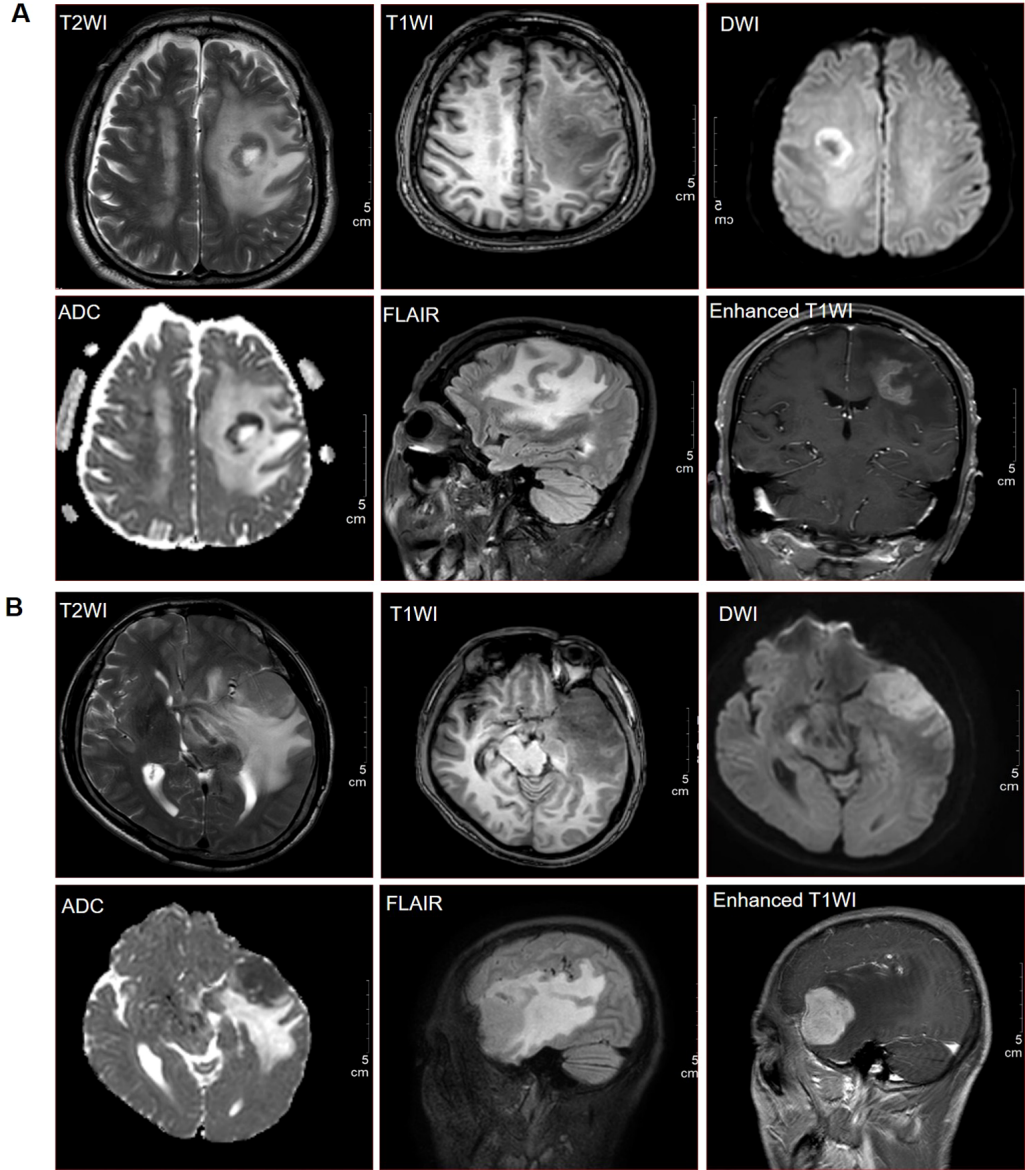
Representative MRI image of hyperintense lesions in PCNSL **(A)** and TDLs **(B)**. **(A)** PCNSL MRI image showing hypointense in lesions in the left frontal lobe, with peripheral edema on T2WI sequence; T1WI sequence showing hypointense in lesions; DWI sequence showing U-shaped hyperintense; ADC sequence showing hypointense in lesions; FLAIR sequence showing hyperintense, with peripheral edema; enhanced T1WI sequence showing U-shaped hyperintense. **(B)** TDLs MRI image showing hypointense in lesions in the left temporal pole on T2WI sequence; T1WI sequence showing hypointense in lesions; DWI sequence showing mild diffused elevated signal in lesions; ADC sequence showing hypointense in lesions; FLAIR sequence showing isointense, with peripheral edema; enhanced T1WI sequence showing hyperintense, with peripheral edema.

Upon MRI scans, most brain lesions of TDLs and PCNSL showed hypointense on T2WI and T1WI sequences. On diffusion-weighted imaging (DWI) sequences, brain lesions in PCNSL showed U-shaped hyperintense, while lesions in TDLs showed mild diffuse hyperintense. Both PCNSL and TDLs lesions presented hypointense on apparent diffusion coefficient (ADC) sequences. For fluid-attenuated inversion recovery (FLAIR) sequences, PCNSL lesions showed obvious hyperintense and perifocal edema, while TDL lesions showed isointense with partial perifocal edema. On the contrast-enhanced T1WI sequences, lesions in PCNSL and TDL showed hyperintense, with PCNSL lesions exhibiting an obvious C-like shape. The representative typical MRI images are shown in **Figure 1**.

### 3.3 Pathological features of PCNSL and TDL

**Figure 2:**
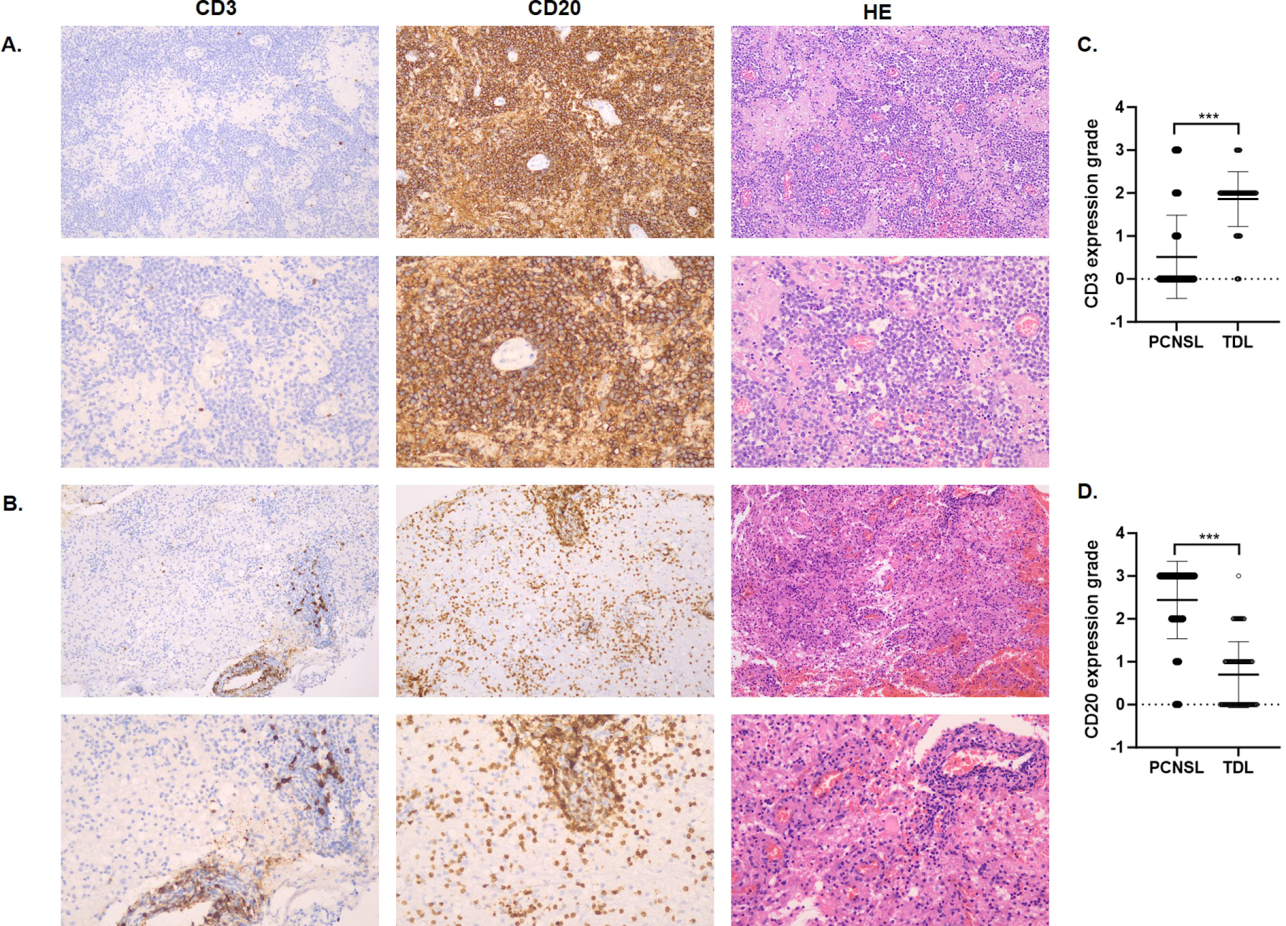
Pathological features of PCNSL and TDL. **(A)** IHC staining of CD3, CD20, and HE staining of PCNSL. **(B)** IHC staining of CD3, CD20, and HE staining of TDLs. **(C, D)** The expression grades of CD3 and CD20 between PCNSL and TDLs groups. Data are presented as mean ± SD, ***p<0.001. Scale bar =50 or 200 μm.

We also investigated the differences in the pathological features between PCNSL and TDLs. Immunohistochemical (IHC) staining for CD3 and CD20, along with hematoxylin–eosin (HE) staining, was performed on surgical/biopsy specimens from all 97 PCNSL and 86 TDLs patients included in this study, with no missing samples. The typical pathological feature of PCNSL is that the tumor cells are arranged in a cuff-like pattern around blood vessels. The tumor cells are basically uniform in size and morphology. Lymphoid follicles are rare in the tumors. IHC staining of the PCNSL tissues showed that CD20 was strongly positive, while CD3 was negative. TDLs are pathologically characterized by demyelination and extensive infiltration of inflammatory cells and macrophages in both gray and white matter, with telangiectasia and hemorrhage. All TDLs patients in the present study showed marked perivascular inflammatory lymphocyte cuffs. The CD3 positivity rate in PCNSL was significantly lower than that in TDLs (25.77% vs. 94.19%), whereas the CD20 positivity rate in PCNSL was significantly higher than that in TDLs (91.75% vs. 52.32%). CD20 expression grade in the PCNSL group was significantly higher than that in the TDL group (p < 0.001), while the CD3 expression grade in the PCNSL group was significantly lower than that in the TDL group (p < 0.001) (**Figures 2C, D**). Representative typical pathological images are shown in **Figures 2A, B**).

### 3.4 Cerebrospinal fluid and serum analyses

**Figure 3:**
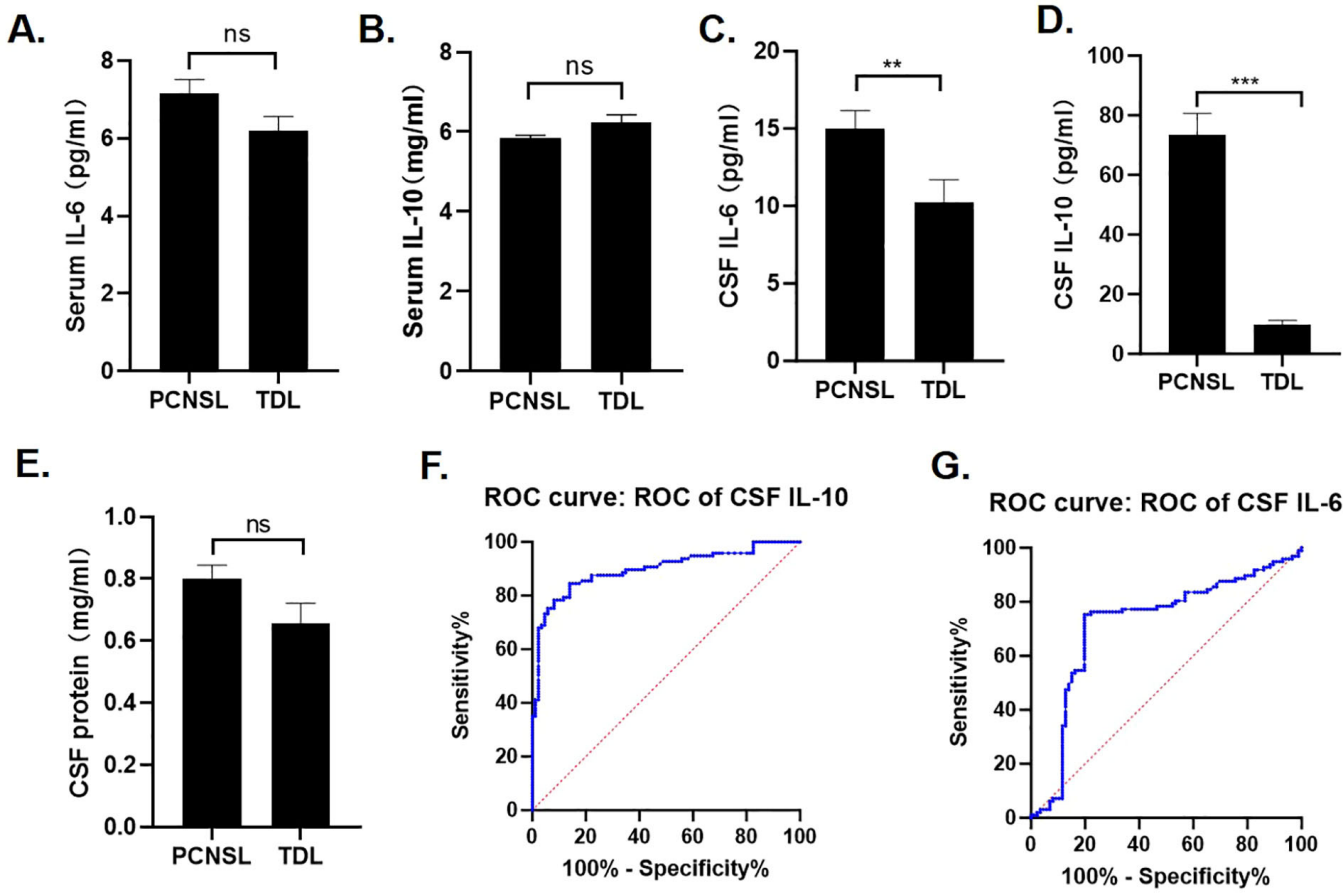
Difference of markers in CSF and serum between the PCNSL and TDL patients. **(A, B)** Serum IL-6 and IL-10 levels were detected by ELISA. **(C, D)** CSF IL-6 and IL-10 levels were detected by ELISA. **(E)** CSF protein levels were determined by using the protein assay kit. **(F, G)** Receiver operating characteristic (ROC) curves constructed for CSF IL-10 and IL-6. n=97 in the PCNSL group, and n=86 in the TDL group. Data are presented as mean ± SD, **p<0.01, ***p<0.001.

IL-6 and IL-10 are known to be closely associated with lymphocyte immunology, and their abnormal expression has been linked to poor prognosis in non-Hodgkin lymphoma (17, 18). Elevated CSF IL−10 levels have also been reported in multiple sclerosis and neuromyelitis optica spectrum disorder (19). Increased CSF IL−6 levels are frequently observed in multiple sclerosis and other inflammatory neurological diseases (20). However, conflicting evidence indicates that CSF IL-6 and IL-10 levels may remain within normal ranges in multiple sclerosis (21). So, we investigated the differences in IL-10 and IL-6 levels in the serum and CSF between patients with PCNSL and TDLs. We found that there was no statistical differences in IL-10 and IL-6 levels in the serum between patients with PCNSL and TDLs (**Figures 3A, B**). The IL-6 and IL-10 levels in the CSF of PCNSL patients were significantly higher than those of TDLs patients (**Figures 3C, D**). High CSF protein concentration was adversely related to the prognosis of PCNSL (22). We measured the protein concentration in the CSF of patients with PCNSL and TDLs. Although the CSF protein concentration showed an increasing tendency in PCNSL patients, there’s no statistical difference in the CSF protein level between patients with PCNSL and TDLs (**Figure 3E**).

Then we constructed ROC curves to discriminate the potential use of CSF IL-6 and IL-10 levels for the diagnosis of PCNSL (**Figure 3F**). CSF IL-10 showed excellent (p < 0.001) discriminator power for the diagnosis of PCNSL (cut-off ≥ 9.095 pg/mL; sensitivity 84.54%, specificity 86.05%; Youden index = 0.706) as the AUC was 0.898 ± 0.023 (95% CI = 0.852–0.945), followed by CSF IL-6 (p < 0.001) with an AUC of 0.719 ± 0.040 (95% CI = 0.640–0.799; cut-off ≥ 8.135 pg/mL; sensitivity 75.26%, specificity 80.23%; Youden index = 0.555).

## 4 Discussion

PCNSL is a relatively rare type of non-Hodgkin lymphoma affecting the central nervous system, which mostly occurs in immunocompromised patients (23, 24). The clinical symptoms of the disease are insidious, making it difficult to make a differential diagnosis based on clinical characteristics alone. TDL is a rare demyelinating disease of the central nervous system, with the destruction and demyelination of nerve fibers as its main pathological features (25). Some imaging and clinical features of TDL can be very similar to those of PCNSL, leading to misdiagnosis and inappropriate treatment of TDL and PCNSL (26, 27).

PCNSL often occurs in elderly patients (28), while TDLs often occur in middle age (29). In a study of 480 patients with TDLs, PCNSL, tumefactive primary angiitis of the central nervous system, and brain gliomas, the average age of PCNSL patients is significantly higher than that of TDLs patients (57.60 ± 13.39 vs 37.29 ± 14.06) (30). Consistently, we found that the average age of PCNSL patients was markedly higher than the TDLs patients (59.28 ± 10.23 vs 36.28 ± 8.11). The main initial symptoms of PCNSL include cognitive dysfunction, headache, reduced vision, and weakness, and seizures (31). In the present study, there was no difference in initial symptoms between PCNSL and TDLs. The main initial symptoms of TDLs were headache, limb numbness, and reduced vision (32). In addition, the imaging findings of TDLs mainly shows intracranial space-occupying lesions, which are easy to be misdiagnosed as brain tumors, including gliomas and PCNSL (32).

Neuroimaging features of PCNSL on MRI can be very variable. In the present study, brain lesions of TDLs and PCNSL mainly showed hypointense on T2WI, T1WI, and ADC images, making no room for differentiating between PCNSL and TDLs on these points. PCNSL shows hypointense or isointense on T1WI, and hypointense, isointense or hyperintense on T2WI (33–38). TDLs often show hypointense on T1WI, but both hypointense and hyperintense could be observed on T2WI (39, 40). DWI measures the diffusion of water molecules in biological tissues (41). Diffusion within the tumor is considered a surrogate marker of tumor cellularity because intact cells constitute a barrier to water diffusion (41). Because CNS lymphomas are highly cellular tumors, water diffusion is often restricted, making them appear hyperintense on DWI images (41–44). Consistently, we found that brain lesions in PCNSL showed hyperintense, while lesions in TDLs showed slightly diffuse hyperintense on DWI images. Wang et al. (38) reported that the specificity and positive predictive value of FLAIR signal intensity on PCNSL could be 100% when differentiated from glioblastoma. We found that lesions in PCNSL showed obvious hyperintense on FLAIR sequences with perifocal edema, while TDLs lesions showed isointense with partial perifocal edema. The most important imaging feature of PCNSL is its performance in enhanced MRI. PCNSL has a more significant damage to the blood-brain barrier (45). Therefore, PCNSL usually tends to present a uniformly strengthened signal in the contrast-enhanced image (46). We found that both lesions in PCNSL and TDLs showed hyperintense on the contrast-enhanced T1WI sequences, with PCNSL lesions exhibiting an obvious C-like shape, which is consistent with previous report (47).

Typical histopathological characteristics of PCNSL include diffuse infiltrative and highly proliferating lymphoma cells with angiocentric growth patterns (31), and EBV-positive PCNSL often exhibits larger areas of necrosis (48). The basic histopathologic features of active-stage TDLs consist of demyelinated areas with relative axonal sparing, inflammatory infiltrates mainly composed of myelin-containing foamy macrophages, perivascular lymphocytes, and reactive astrocytes that may contain multiple nuclei (Creutzfeldt–Peters cells), as well as telangiectasia and hemorrhage (49). In the present study, we found that the tumor cells were arranged in a cuff-like pattern surrounding blood vessels in the PCNSL tissue, and little lymphoid follicles were rarely observed in the tumors. All TDLs patients enrolled in the present study exhibited prominent perivascular inflammatory lymphocyte cuffs. The pan B-cell markers CD19, CD20, and CD79a are universally positive, whereas CD3, a pan T-cell marker, is negative in PCNSL (50), while CD3(+) T-cells could be identified within the demyelinated foci (51), making it a reliable marker to distinguish TDL from PCNSL. Consistent with previous reports, PCNSL tissues showed strong positive CD20 expression and negative CD3 expression. In TDL tissues, both CD20 and CD3 were positive; however, CD20 expression was weaker and CD3 expression was stronger compared with those in PCNSL tissues.

Diagnosis of PCNSL is challenging, and although brain biopsy remains the gold standard, finding a less invasive source of lymphomatous biomarkers is still of vital importance. Serum IL-10 and IL-6 levels have been reported to correlate with prognosis in non-Hodgkin’s lymphoma (52). However, no statistical differences were observed in IL-10 and IL-6 levels in the serum between the patients with PCNSL and TDLs in the present study. The IL-6 and IL-10 levels in the CSF of PCNSL patients were significantly higher than those with TDLs. The lack of significant differences in serum IL-6 and IL-10 levels between PCNSL and TDL groups can be mainly explained by the blocking effect of the blood–brain barrier (BBB). Lesions in PCNSL are confined to the central nervous system, and IL-6/IL-10 secreted by tumor cells accumulate primarily in the cerebrospinal fluid (CSF), with limited ability to cross the BBB into the peripheral circulation, resulting in no obvious elevation in serum levels. Similarly, inflammation in TDL is predominantly localized in the central nervous system, without specific changes in peripheral inflammatory cytokines. Our findings are consistent with a previous study which reported that low levels of IL-6 were detected in most serum from MS and NIND patients (20). ROC curve analysis demonstrated that cerebrospinal fluid (CSF) IL-10 and IL-6 levels exhibit considerable diagnostic value for differentiating PCNSL from TDL. The optimal cut-off values for identifying PCNSL were determined as IL-6 ≥ 8.135 pg/mL and IL-10 ≥ 9.095 pg/mL, respectively. Consistent with our findings, a previous study reported a cut-off value of 9.5 pg/mL for CSF IL-10, which yielded a sensitivity of 0.71 and a specificity of 1.00 for PCNSL diagnosis; for CSF IL-6, the same study identified a cut-off of 4.0 pg/mL with a sensitivity of 0.77 and a specificity of 0.63 (53). The high concordance in the diagnostic performance of CSF IL-10 between the present study and prior research further validates that CSF IL-10 is a reliable differential diagnostic marker for PCNSL. High CSF protein concentration was adversely related to prognosis of PCNSL (22). It has been reported that more than half of PCNSL cases present elevated CSF protein levels (54–56). We found that the CSF protein levels tended to be higher in PCNSL patients, with no statistical difference between the two groups. The lack of a significance difference in CSF protein levels between PCNSL and TDLs may be attributed to variations in disease stage and the limited sample size. Further investigation with a larger patient cohort is still needed.

## 5 Conclusion

Distinguishing sentinel lesions of PCNSL and TDLs at an early disease stage remains challenging. In this observational study, we reported the differences between PCNSL and TDLs in clinical features, neuroimaging, pathological characteristics, and the biomarkers. Specifically, variations in onset age, MRI findings on DWI and FLAIR sequence, tissues expression of CD20 and CD3, and CSF IL-6 and IL-10 levels were observed between the two groups. These findings may provide supportive information for differential consideration; however, their diagnostic utility requires further validation in larger, prospective studies.

## Data Availability

The datasets presented in this study can be found in online repositories. The names of the repository/repositories and accession number(s) can be found in the article/supplementary material.
